# Human scalp evoked potentials related to the fusion between a sound source and its simulated reflection

**DOI:** 10.1371/journal.pone.0209173

**Published:** 2019-01-09

**Authors:** Ying Huang, Hao Lu, Liang Li

**Affiliations:** 1 Special Laboratory Primate Neurobiology, Leibniz Institute for Neurobiology, Magdeburg, Germany; 2 School of Psychological and Cognitive Sciences, Peking University, Beijing, China; 3 Beijing Key Laboratory of Behavior and Mental Health, Peking University, Beijing, China; 4 Speech and Hearing Research Center, Peking University, Beijing, China; 5 Key Laboratory on Machine Perception (Ministry of Education), Peking University, Beijing, China; 6 Center for Behavioral Brain Sciences, Otto-von-Guericke-University, Magdeburg, Germany; 7 Beijing Institute for Brain Disorders, Beijing, China; Harvard Medical School, UNITED STATES

## Abstract

The auditory system needs to fuse the direct wave (*lead*) from a sound source and its time-delayed reflections (*lag*) to achieve a single sound image perception. This lead-lag fusion plays crucial roles in auditory processing in reverberant environments. Here, we investigated neural correlates of the lead-lag fusion by tracking human cortical potentials evoked by a break in the correlation (*BIC*) between the lead and lag when the time delay between the two was 0, 2, or 4 ms. The BIC evoked a scalp potential consisting of an N1 and a P2 component. Both components were modulated by the delay. The effects of the delay on the amplitude of the two components were similar, an increase of the delay resulting in a decrease of the amplitude. In contrast, the delay differently modulated the latency of the two components, an increase of the delay resulting in an increase of the P2 latency but not an increase of the N1 latency. Similar to the P2 latency, the reaction time for subjective detection of the BIC also increased with the delay. These findings suggest that both the N1 and the P2 evoked by the BIC are neural correlates of the lead-lag fusion and that, relative to the N1, the P2 may be more closely related to listeners’ perception of the fusion. Our study thus provides a neurophysiological and objective approach for investigating the fusion between the direct sound wave from a sound source and its reflections.

## Introduction

In reverberant environments, listeners confront not only direct waves from a sound source but also numerous time-delayed, filtered reflections from nearby surfaces such as walls and other objects. The auditory system needs to fuse the direct wave (*lead*) and its reflections (*lag*) to achieve a single sound image perception (*the precedence effect*) [1–4]. This lead-lag fusion is crucial for the localization of the sound image and for the perception of the sound image including its diffuseness, loudness as well as pitch [3], thus playing critical roles in auditory processing in noisy and reverberant environments [5–8]. It has been shown that the strength of the fusion is a function of the time delay between the onsets of the lead and lag [3, 6]. For example, for delays ≤ 4 ms, the lead and lag are fused into a single sound image whose diffuseness increases with the delay. For longer delays, e.g., > 10 ms, the lead and lag are perceived as two sequential, spatially-separated sound images, indicating a breakdown of the fusion. The strength of the fusion depends also on the similarity (measured as *correlation*) between the waveforms of the lead and lag, decreasing with a decrease of the correlation [3, 9, 10]. For example, when the lead and lag are correlated (correlation = 1), listeners experience the aforementioned “classic” fusion phenomenon. In contrast, when the lead and lag are uncorrelated (correlation = 0), the fusion breaks down and two sound images are perceived, regardless of the delay.

For delays > 0 ms, the successful lead-lag fusion requires storing the fine structure information of the lead in “primitive” auditory memory [6–8, 11] for instantaneously computing the correlation between the lead and lag as well as for integrating the computation results over time [12]. The stored information of the lead decays over time, resulting in a decrease of the correlation between the lead and lag at the neural level and thus a decrease of the fusion at the perceptual level. When the delay increases to a certain value, it becomes difficult for listeners to discriminate the sound image(s) resulted from the fusion of physically correlated lead and lag from the sound image(s) resulted from the fusion of physically uncorrelated lead and lag. Therefore, one way to quantify the strength of the lead-lag fusion at a certain delay is to measure the detection of a change in the correlation between the lead and lag from 1 to 0 and then returning back to 1 [7, 8, 11, 13, 14]. This change in the correlation has been termed *break in correlation* (BIC) in previous studies [15–17]. The strength of the lead-lag fusion quantified in this way utilizing white noises reflects listeners’ performance of speech perception in simulated noisy and reverberant environments [7, 8, 11].

Although the psychoacoustics associated with this lead-lag fusion have been well documented [7, 8, 11, 13, 14], its underlying neural mechanisms in human listeners are poorly understood. Our previous study [18] has shown that the BIC can evoke a scalp potential consisting of an N1 and a P2 component and that the absolute difference in the peak amplitude of these two components (i.e., the N1-P2 peak-to-peak amplitude) decreases with an increase of the delay. These observations suggest that the N1 and the P2 evoked by the BIC may be neural correlates of the lead-lag fusion. Nevertheless, the N1 and the P2 were analyzed as a complex rather than as separate components in the study [18]. Therefore, it remains still unclear whether the N1, or the P2, or both are neural correlates of the lead-lag fusion. It is also not clear whether these two components are related to listeners’ perception of the fusion or not and which component is more closely related to the perception. To address these issues, we separately analyzed the N1 and the P2 evoked by the BIC in this study. We took advantages of our previous EEG studies [18] by reanalyzing the EEG dataset and conducted a new EEG experiment as well as a new psychoacoustic experiment.

## Materials and methods

### Subjects

Thirty university students (18–29 years old, mean age = 22.0 years, 19 females, 11 males) participated in this study. All of them are right-handed, determined by the hand writing individual signatures. They all had normal (≤ 25 dB) and balanced (≤ 20 dB difference between the two ears) pure-tone hearing thresholds at frequencies from 0.125 to 8 kHz, which were confirmed by an audiometer. They gave their written informed consent to participate the study and were paid a modest stipend for the participation. Twelve subjects participated in EEG experiment 1. Part of the data obtained from this EEG experiment has been published [18]. Six subjects participated in EEG experiment 2. The other twelve subjects participated in the psychoacoustic experiment. Data obtained from one subject in the psychoacoustic experiment was excluded from analyses because this subject did not properly follow experimental instructions. The Committee for Protecting Human and Animal Subjects of the School of Psychological and Cognitive Sciences at Peking University has approved the study.

### Stimuli and apparatus

**Fig 1:**
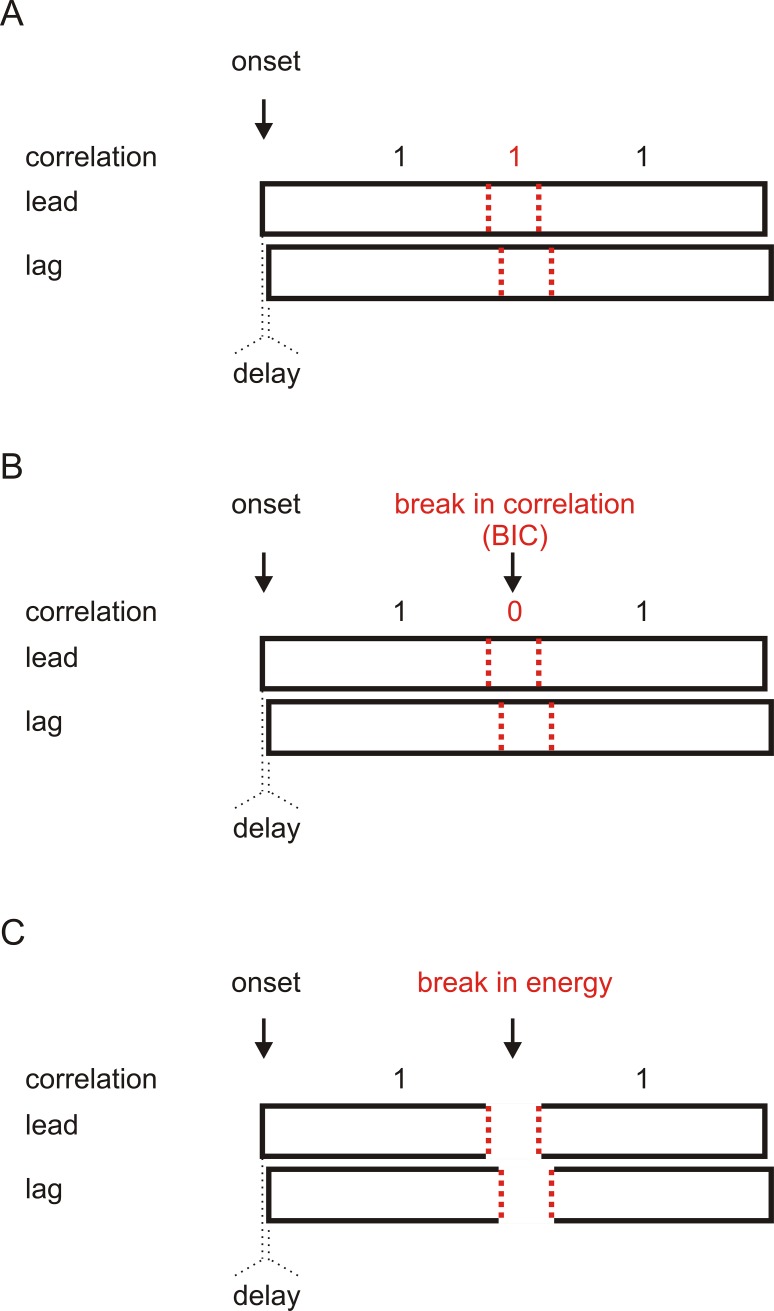
Illustration of the auditory stimuli. The schematic of a pair of identical white noises without a break in correlation or a break in energy in the temporal middle of the noises (A), with a break in correlation (B), or with a break in energy (C). For each pair of noises, one was presented as the lead and the other as the lag, separated by a delay of 0, 2, 4, or 8 ms. The dynamic correlation between the lead and lag during noise presentation is provided.

Three types of stimuli were used (Fig 1). The first type was a pair of identical Gaussian steady-state wideband white noises generated utilizing the “randn()” function in the MATLAB function library (MathWorks; 10 kHz low-pass filtered, 48-kHz sampling rate, and 16-bit amplitude quantization), with one noise presented as the lead and the other as the lag. The correlation between the lead and lag was always 1 during the presentation of the noises (Fig 1A). The second and third types of stimuli were generated based on the first type, by introducing either a break in correlation (BIC; Fig 1B) or a break in energy (Fig 1C) in the central part the two noises. To introduce a BIC, the 200-ms central part of the lead was replaced by a 200-ms noise fragment which was uncorrelated with the corresponding part of the lag. The correlation between the lead and lag therefore changed from 1 to a value near 0 (not 0 but <0.0045, because two randomly generated noise fragments are not necessarily orthogonal) and then back to 1 during noise presentation (Fig 1B). The replacement of the noise fragment changed neither the spectrum nor the sound pressure level of the noise. To introduce a break in energy, the 200-ms central part of both the lead and lag was replaced by a 200-ms silent gap (including 5-ms fall/rise times to avoid spectrum splatter). The duration of the BIC and the break in energy was fixed at 200 ms according to our previous study [7]. The wideband noises could be then band-pass filtered to obtain narrowband noises with a center frequency of 400 or 800 Hz (bandwidth = 1/3 octaves). In the EEG experiments, the duration of the noises was 2000 ms (including 30-ms rise/fall times), and the BIC or the break in energy started from 900 ms after the onset of the lead-lag pair. In the psychoacoustic experiment, the duration of the noises was 8000 ms (including 30-ms rise/fall times), and the BIC or the break in energy started randomly from 3000 to 5000 ms after the onset of the lead-lag pair.

For each lead-lag pair, the lead and lag were separated by a delay of 0, 2, 4, or 8 ms, depending on the experiments. The lead and lag were presented to subjects through two tube phones in the EEG experiments, with the lead in the left ear and the lag in the right ear, or through two headphones (HD 265 linear, Sennheiser, Germany) in the psychoacoustic experiment, with the lead in the left or in the right ear. Tube phones or headphones, instead of loudspeakers, were used to present the noises to avoid potential spectral changes induced by the BIC [19]. The noises were transferred using the Creative Sound Blaster (Creative SB Audigy 2 ZS, Creative Technology, Ltd, Singapore) and presented at a sound pressure level of 56–60 dB, calibrated by the Larson Davis Audiometer Calibration and Electroacoustic Testing System (AUDit and System 824, Larson Davis, USA) with “A” weighting.

All experiments were conducted in sound attenuated chambers (EMI Shielded Audiometric Examination Acoustic Suite).

### Procedures

In EEG experiment 1, each lead-lag pair could be a pair of 400-Hz or a pair of 800-Hz narrowband noises. There was either the BIC or the break in energy in the temporal middle of the noise pair. The lead and lag were separated by a delay of 0, 2, or 4 ms. Therefore, overall, there were 12 lead-lag pairs (3 delays × 2 center frequencies × 2 breaks). Each pair with the BIC was presented 120 times and each pair with the break in energy 24 times. Fresh noises were used for each presentation. The overall 864 presentations were divided into 12 blocks. In a block, each pair with the BIC was presented 10 times and each pair with the break in energy 2 times. All pairs were presented in random order with an interval of 1000 ms between adjacent presentations. To ensure that subjects kept their attention on the noises, they were required to press a button in a response box as quickly as possible when they detected the break in energy in a lead-lag pair [20].

In EEG experiment 2, each lead-lag pair was a pair of wideband noises, with the BIC or the break in energy in the temporal middle, or with no breaks in the temporal middle. The delay between the lead and lag was fixed at 0 ms. Therefore, there were 3 lead-lag pairs in total. Each pair was presented 120 times and fresh noises were used for each presentation. The overall 360 presentations were divided into 6 blocks. In a block, each of the three pairs was presented 10 times. Other procedures were identical to those in EEG experiment 1.

In the psychoacoustic experiment, there were three blocks. The reaction time for detecting the BIC, the break in energy, or the onset of the lead-lag pair was measured in separate blocks. In each block, a lead-lag pair could be a pair of 400-Hz or a pair of 800-Hz narrowband noises, with the lead and lag being separated by a delay of 0, 2, 4, or 8 ms. Thus, there were 8 lead-lag pairs (4 delays × 2 center frequencies) in total. Each pair was presented 20 times and fresh noises were used for each presentation. In addition, a sham stimulus was presented 20 times to track the appropriateness of subjects’ behavioral responses, i.e., to track whether subjects gave a behavioral response following the experimental instructions or just randomly. All stimuli were presented in random order with a 2000-ms interval between adjacent presentations. Subjects were asked to press a button on a computer keyboard using their right hand as quickly as possible when they detected the target event. In the blocks for measuring the reaction time for detecting the BIC and the break in energy, the sham stimulus was a lead-lag pair with no breaks in the temporal middle. In the block for measuring the reaction time for detecting the onset of the lead-lag pair, a pair of 8000-ms silent periods was used to track the appropriateness of subjects’ behavioral responses. The order of the three blocks was counter-balanced across the subjects using Latin square.

### Data recordings and analyses

In the EEG experiments, EEG signals were recorded using 64-channel NeuroScan SynAmps (Compumedics Limited, Victoria, Australia). Subjects were instructed to remain alert and fixate a red light in the frontal field when they listened to the noises. During EEG recordings (band-pass filter: 0.05–40 Hz; sampling rate: 1000 Hz), all electrodes were referenced to the head center. Ocular artifacts were corrected offline using the linear regression method [21] built in the Neuroscan Software (Compumedics Limited, Victoria, Australia).

Further preprocessing and analyses were conducted using Matlab (MathWorks). All EEG signals were first high-pass filtered at 1 Hz using a second-order Butterworth filter which was designed using the “butter()” function in the MATLAB function library. The signals were then re-referenced to an average reference. That is, the signals averaged across all channels were subtracted from the signals in individual channels. The signals in each channel were then cut into epochs from -100 to 500 ms relative to the onset of the lead-lag pair or relative to the onset of the BIC in the lead-lag pair. Epochs with signals exceeding ±150 μV were considered being contaminated by artefacts and thus excluded from analyses. All epochs were corrected relative to the baseline that was calculated as the mean amplitude during the period from -100 to 0 ms. The epochs were then averaged to obtain the waveform of the event-evoked potential for each subject, condition, and channel. Data from the nine central channels (F1, FZ, F2, FC1, FCZ, FC2, C1, CZ, and C2) were averaged for further analyses. The group-mean waveform of the event-evoked potential in a certain condition was obtained by averaging the waveforms in the condition across subjects.

We investigated the peak amplitude and peak latency of the N1 and P2 components of the event-evoked potential. For the N1 / P2 component of the potential evoked by the onset of the lead-lag pair (*onset-evoked N1/P2*), the peak latency was the time at which the largest negative / positive potential (*peak amplitude*) was obtained in the temporal window from 80 to 180 ms / from 150 to 250 ms after the onset of the lead-lag pair. For the N1 / P2 component of the potential evoked by the BIC (*BIC-evoked N1/P2*), the peak latency and amplitude were obtained in the window from 100 to 200 ms / from 200 to 350 ms after the onset of the BIC. These windows were visually determined according to the mean waveforms averaged across conditions and subjects.

In the psychoacoustic experiment, the reaction time for detecting the BIC, the break in energy, or the onset of the lead-lag pair was measured and recorded using Matlab (MathWorks).

For EEG experiment 1 and for the psychoacoustic experiment, data obtained from the 400- and 800-Hz narrowband noises were pooled together because results obtained from the two noises exhibited similar patterns.

## Results

In both EEG experiments, subjects kept their attention on the noises. This was evidenced by the observation that the mean hit rate for detecting the break in energy in the lead-lag pair reached up to 98% (SE = 0.5%). In addition, all the subjects reported that they perceived a single-fused noise image under each of the stimulation conditions and that they were able to detect the BIC under the conditions.

### EEG experiment 1

**Fig 2:**
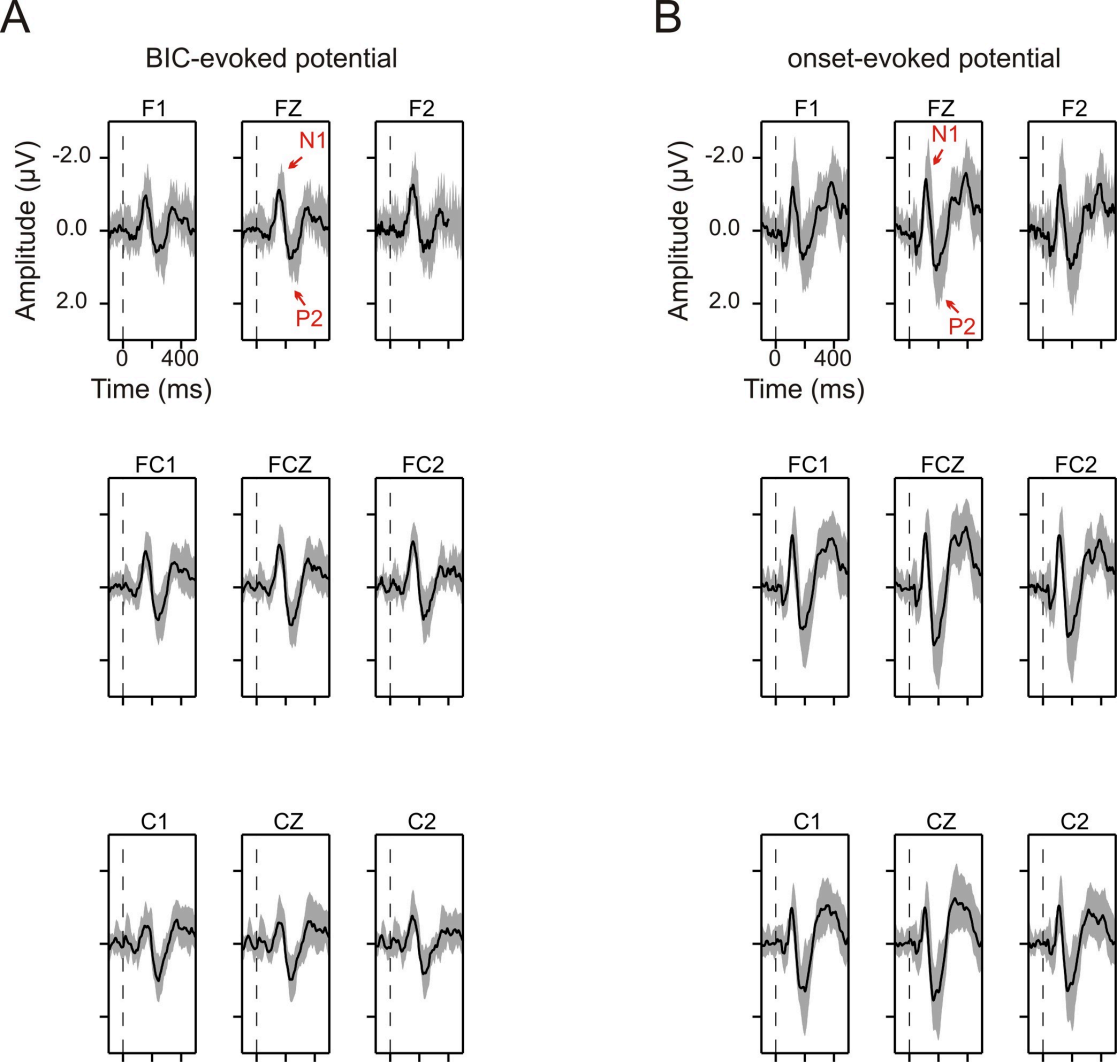
Group-mean evoked potentials on each of the nine central channels when the delay was 0 ms. (A) BIC-evoked potentials on individual channels. In each panel, the black trace represents the group-mean evoked potential averaged across 12 subjects, with data obtained from 400- and 800-Hz narrowband noises being pooled together. The grey area represents the group mean potential ± one standard deviation. The vertical dashed line marks the onset of the BIC. Time is expressed relative to the onset of the BIC. The name of each channel is provided. (B) Onset-evoked potentials on individual channels. Conventions as in panel A.

In this EEG experiment, when the delay was 0 ms, not only the onset of the lead-lag pair but also the BIC in the pair could evoke a scalp potential (*onset-evoked* and *BIC-evoked potential*, *respectively*) on many channels. The BIC-evoked potential consisting of an N1 and a P2 component was observed most obviously on the nine central channels: F1, FZ, F2, FC1, FCZ, FC2, C1, CZ, and C2, and the potential on each of these channels had similar morphology (Fig 2A). Similar observations were obtained for the onset-evoked potential (Fig 2B). The evoked potentials from these nine channels were therefore averaged for further analyses. The averaging was conducted separately for the BIC-evoked and onset-evoked potentials.

**Fig 3:**
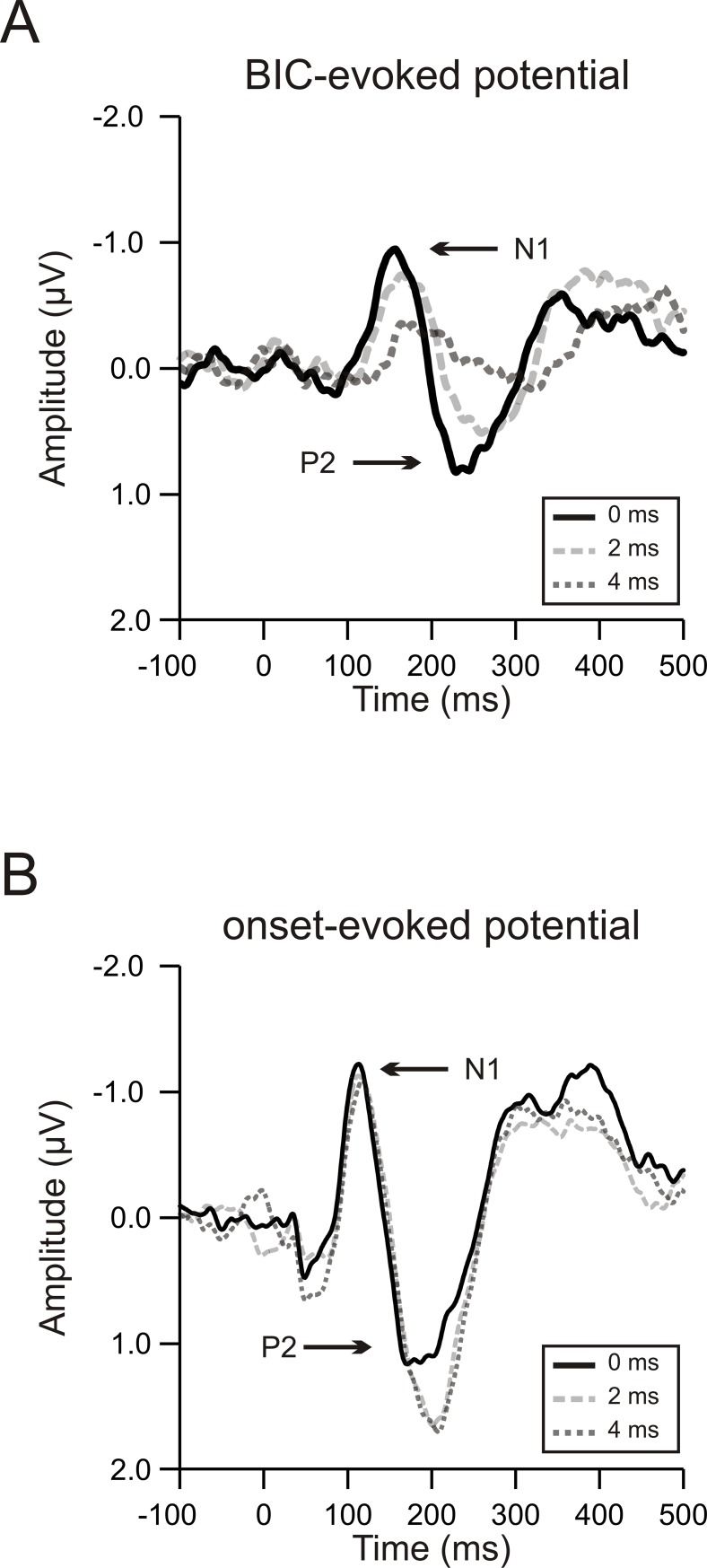
BIC-evoked and onset-evoked potentials at different delays. (A) BIC-evoked potentials when the delay was 0, 2, or 4 ms. Each trace represents the group-mean waveform of the BIC-evoked potential averaged across 12 subjects, with data obtained from 400- and 800-Hz narrowband noises being pooled together. Note that the potential was averaged from the 9 central channels (F1, FZ, F2, FC1, FCZ, FC2, C1, CZ, and C2). Time is expressed relative to the onset of the BIC. (B) Onset-evoked potentials when the delay was 0, 2, or 4 ms. Conventions as in panel A.

Fig 3A shows the group-mean BIC-evoked potentials when the delay was 0, 2, or 4 ms. The peak amplitude of the N1 and the P2 decreased with an increase of the delay, and the peak latency of both components, especially that of the P2, increased with the delay. These observations indicate effects of the delay on the BIC-evoked potential. In contrast, the delay did not affect the onset-evoked potential: neither the peak amplitude nor the peak latency of the N1 and P2 changed with the delay (Fig 3B). Three-way repeated measure ANOVAs were conducted to examine interactions among either the amplitude or the latency of the potential components (N1 and P2), auditory event (the BIC and the onset of the lead-lag pair), and delay (0, 2, and 4 ms). For the amplitude, a significant three-way interaction was obtained [F(2,22) = 30.231, *p* < 0.0001]. For the latency, a significant three-way interaction was not obtained [F(2,22) = 2.194, *p* = 0.135], but significant two-way interactions were obtained between any two of the three factors [component and auditory event: F(1,11) = 5.369, *p* = 0.041; component and delay: F(2,22) = 4.917, *p* = 0.017; auditory event and delay: F(2,22) = 4.294, *p* = 0.027].

**Fig 4:**
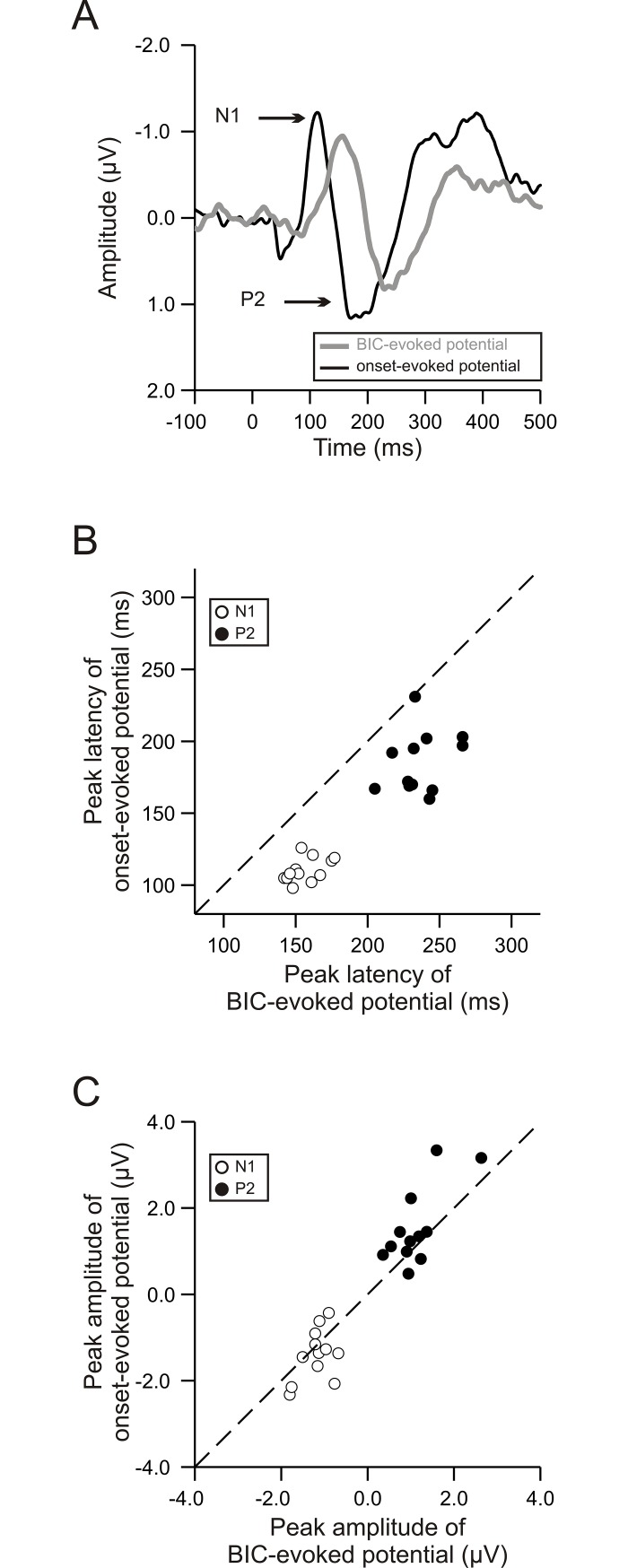
Effects of auditory event on the N1 and P2 components when the delay was 0 ms. (A) Group-mean BIC-evoked (grey) and onset-evoked potentials (black) when the delay was 0 ms. Other conventions as in Fig 3. (B) The peak latency of the onset-evoked potential as a function of the peak latency of the BIC-evoked potential. Open dots represent data for the N1s and filled dots data for the P2s. One dot represents data from one subject. The dashed line marks cases where the two latencies are equal. (C) The peak amplitude of the onset-evoked potential as a function of the peak amplitude of the BIC-evoked potential. Conventions as in panel B.

Specifically, when the delay was 0 ms, the BIC-evoked potential had a significantly longer latency than the onset-evoked potential (*p* < 0.0001, paired-sample t test; compare the grey and black traces in Fig 4A). This observation was obtained from each of the 12 subjects (dots in Fig 4B), for both the N1 (open dots) and the P2 component (filled dots). Relative to the onset-evoked potential, the BIC-evoked potential was delayed on average by ~50 ms, with a slightly longer delay for the P2 than for the N1 (51 vs. 45 ms). In addition, the peak amplitude of the BIC-evoked P2 was smaller than that of the onset-evoked P2 (*p* = 0.042, paired-sample t test; filled dots in Fig 4C). No significant difference in the peak amplitude was observed between the BIC-evoked and the onset-evoked N1 (*p* = 0.186; open dots).

**Fig 5:**
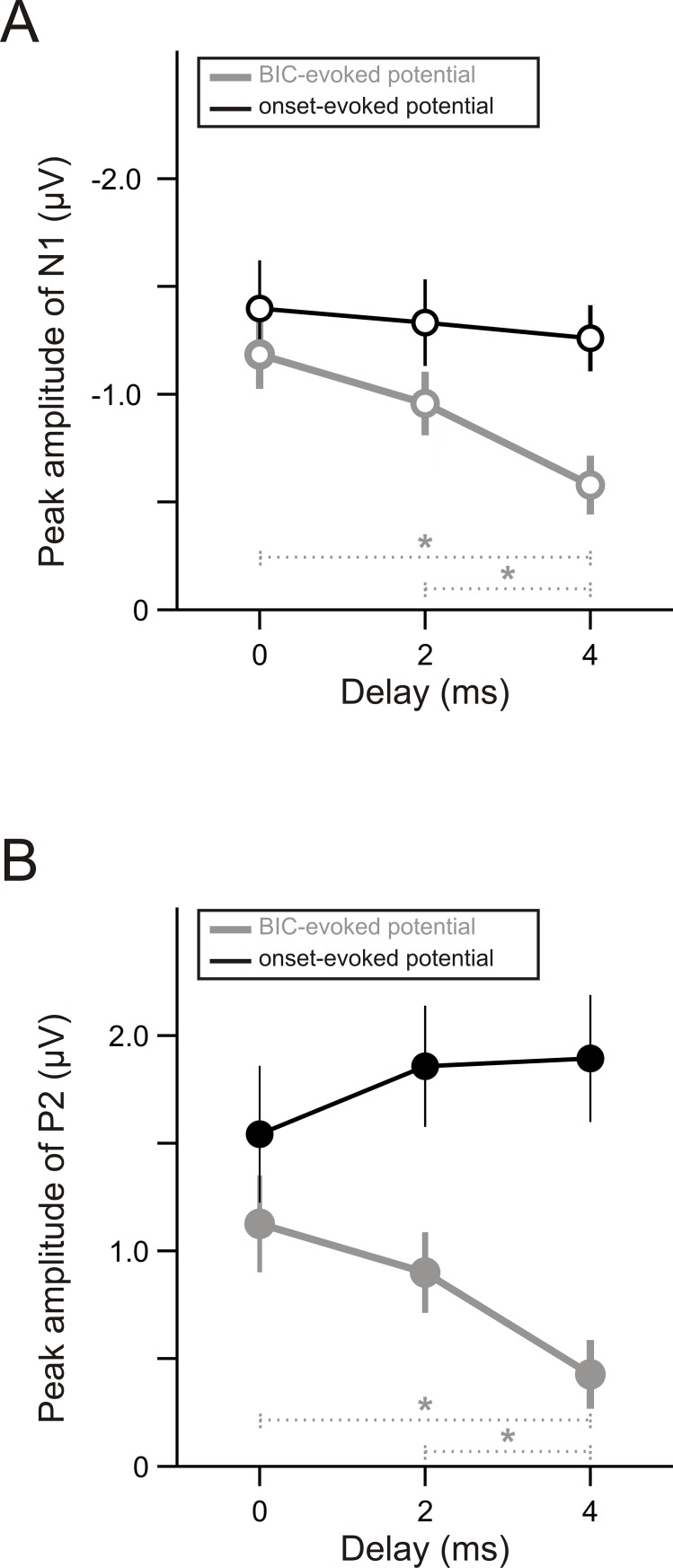
Effects of delay on the amplitude of BIC-evoked and onset-evoked potentials. (A) The peak amplitude of the BIC-evoked (grey) and onset-evoked N1 (black) when the delay was 0, 2, or 4 ms. Error bars represent one time standard error across subjects. * indicates a significant (*p* < 0.05/3) difference between the two conditions. (B) The peak amplitude of the BIC-evoked (grey) and onset-evoked P2 (black) when the delay was 0, 2, or 4 ms. Other conventions as in panel A.

We next investigated effects of the delay on the peak amplitude of the BIC-evoked N1 and P2. One-way repeated measure ANOVAs revealed significant main effects of the delay on the amplitude of both the N1 [Fig 5A, grey dots; F(2,22) = 14.278, *p* = 0.0001] and the P2 [Fig 5B, grey dots; F(2,22) = 17.151, *p* < 0.0001]. The effects of the delay on the amplitude of these two components exhibited similar time courses. Specifically, post-hoc tests showed that the amplitude of both the N1 and the P2 decreased slightly but not significantly (*p* > 0.05/3) when the delay was increased from 0 to 2 ms. The amplitude continued to decrease when the delay was increased further to 4 ms, resulting in significant differences in the amplitude between the 4-ms and the 2-ms delay (*p* = 0.008 for N1; *p* = 0.002 for P2) as well as significant differences between the 4-ms and the 0-ms delay (*p* <0.0001 for N1 and for P2). In contrast to the BIC-evoked N1 and P2, the peak amplitude of the onset-evoked N1 and P2 did not decrease with an increase of the delay: the main effect of the delay on the N1 amplitude was not significant (Fig 5A, black dots), and the P2 amplitude even increased slightly with an increase of the delay (Fig 5B, black dots).

**Fig 6:**
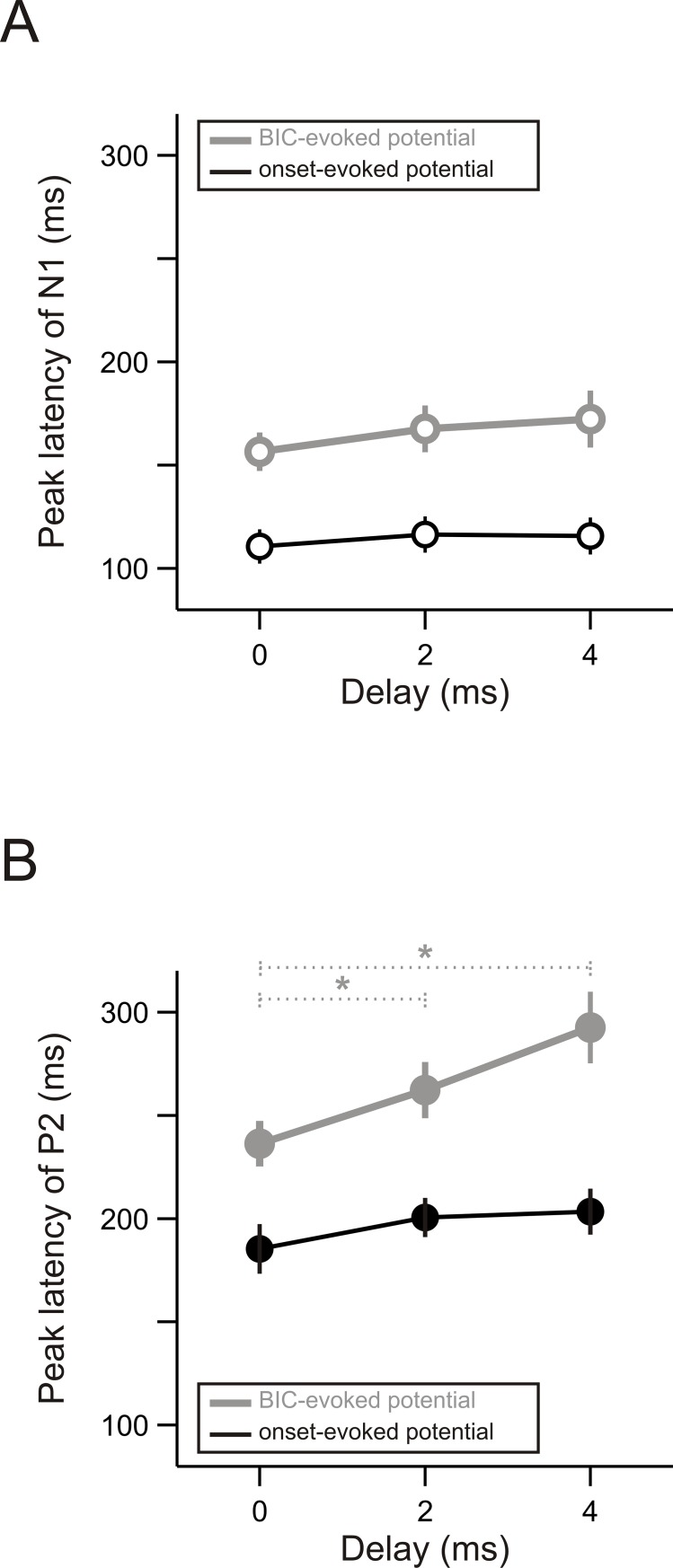
Effects of delay on the latency of BIC-evoked and onset-evoked potentials. (A) The peak latency of the BIC-evoked (grey) and onset-evoked N1 (black) when the delay was 0, 2, or 4 ms. Other conventions as in Fig 5. (B) The peak latency of the BIC-evoked (grey) and onset-evoked P2 (black) when the delay was 0, 2, or 4 ms. Other conventions as in panel A.

The delay also modulated the latency of the BIC-evoked potential. Interestingly, such a modulation of the latency was observed only for the P2 [Fig 6B, grey dots; F(2,22) = 10.45, *p* = 0.006, one-way repeated ANOVA] but not for the N1 [Fig 6A, grey dots; F(2,22) = 1.88, *p* = 0.18]. Further post-hoc tests showed that the P2 latency increased significantly when the delay was increased from 0 ms to 2 ms (*p* = 0.010, paired-sample t test) or from 0 to 4 ms (*p* = 0.001). In contrast to the BIC-evoked potential, no significant effects of the delay were obtained for the latency of the onset-evoked potential: neither the N1 latency nor the P2 latency differed significantly between any two of the three delay conditions (*p* > 0.05/3, paired-sample t test).

### EEG experiment 2

**Fig 7:**
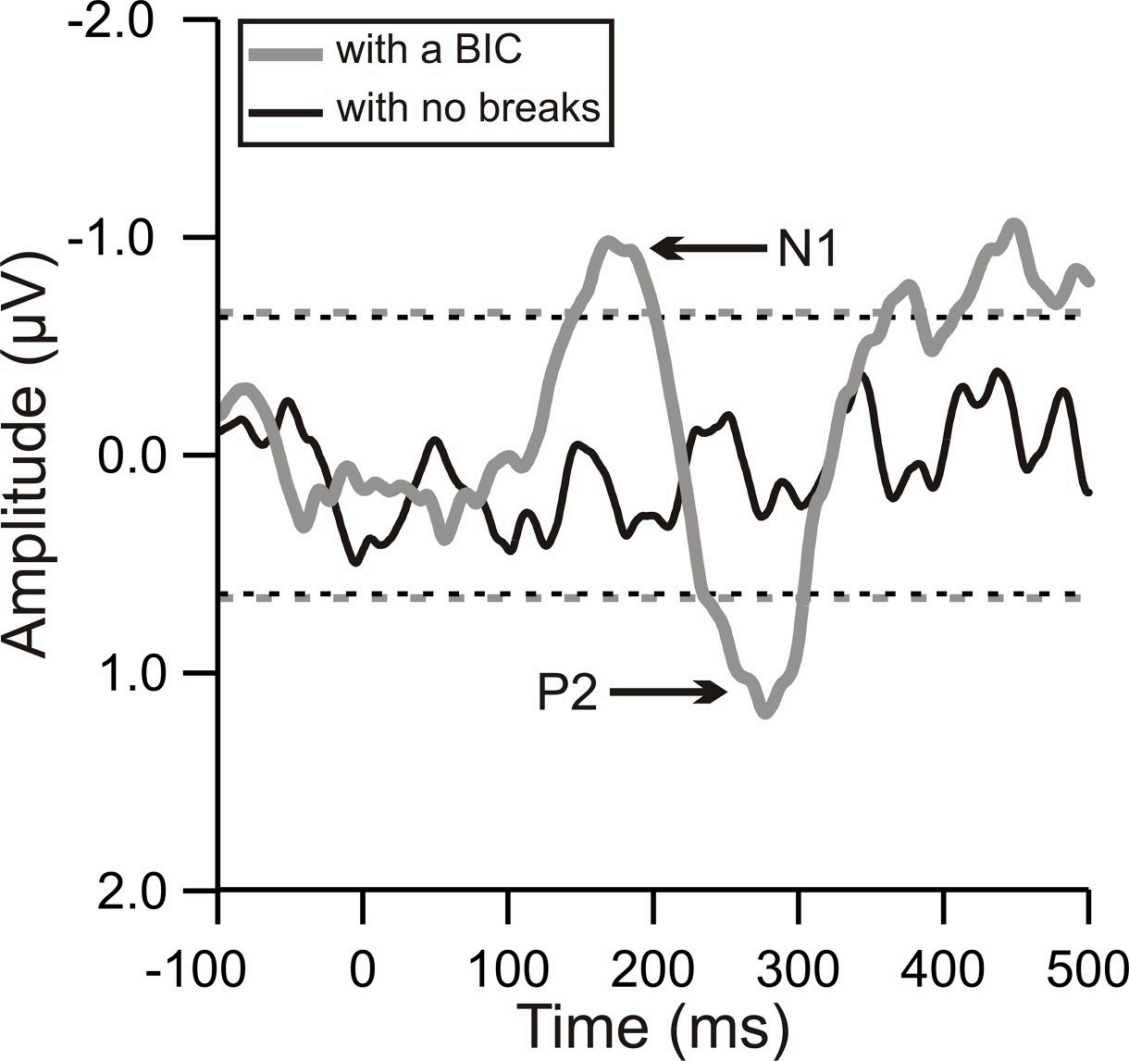
Scalp potentials during the presentation of the lead-lag pair with the BIC or the lead-lag pair with no breaks. The grey trace represents the group-mean waveform of the potential during the presentation of the lead-lag pair with the BIC and the black trace the potential during the presentation of the pair with no breaks. Note that the potentials were averaged across 6 subjects and from the 9 central channels (F1, FZ, F2, FC1, FCZ, FC2, C1, CZ, and C2). Time is expressed relative to the 900 ms after the onset of the lead-lag pair. The dashed lines mark the mean amplitude of the potential during the period from -100 to 0 ms ± three standard deviations.

In EEG experiment 1, the BIC always started from 900 ms after the onset of the lead-lag pair. It is therefore possible that the observed BIC-evoked potential was due to subjects’ temporal expectation rather than due to the occurrence of the BIC. To disentangle these possibilities, we analyzed the EEG signals recorded during EEG experiment 2 in which the lead-lag pair with the BIC and the pair with no breaks were randomly presented to the subjects. Similar to EEG experiment 1, the BIC in EEG experiment 2 always started from 900 ms after the onset of the lead-lag pair. If the BIC-evoked potentials observed in EEG experiment 1 were due to subjects’ temporal expectation, we would observed in EEG experiment 2 evoked potentials similarly during the presentation of the lead-lag pair with the BIC and during the presentation of the pair with no breaks. Nevertheless, such a similarity of the potentials was not obtained. Specifically, when we analyzed the EEG signals ~900 ms after the onset of the lead-lag pairs, we observed an evoked potential only during the presentation of the lead-pair with the BIC but not during the presentation of the pair with no breaks (compare the grey and black traces in Fig 7). These results suggest that subjects’ temporal expectation could not explain the evoked potential observed during the presentation of the lead-lag pair with the BIC, i.e., could not explain the BIC-evoked potential in EEG experiment 2. Based on these findings obtained in EEG experiment 2, we thus conclude that the BIC-evoked potentials observed in EEG experiment 1 were also not due to subjects’ temporal expectation but instead were due to the occurrence of the BIC.

### Psychoacoustic experiment

**Fig 8:**
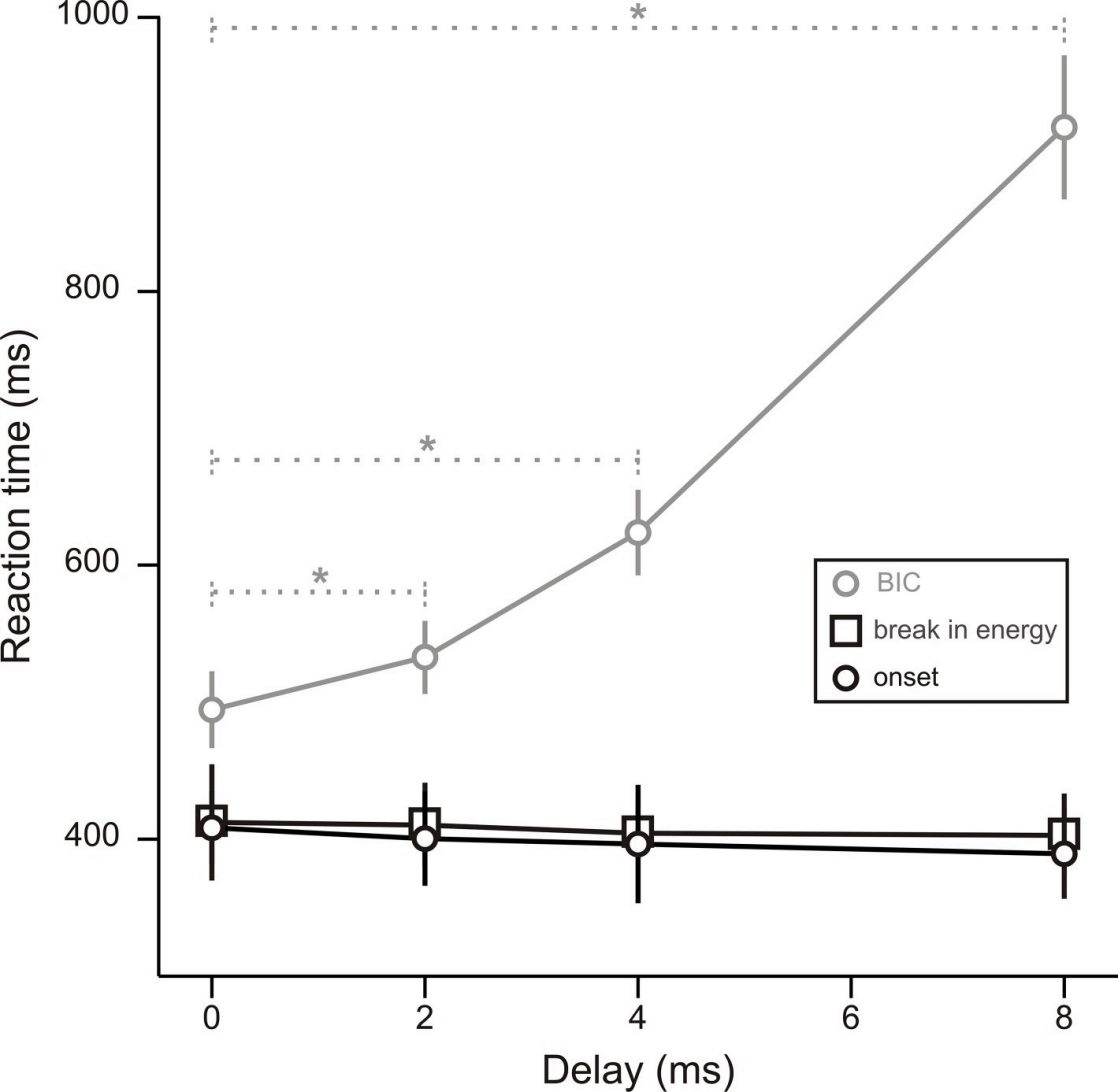
Effects of delay on the reaction time for subjective detection of auditory events. The reaction time for detecting the BIC (grey dots), the break in energy (squares), or the onset of the lead-lag pair (black dots) when the delay was 0, 2, 4, or 8 ms. Error bars represent one time standard error across subjects. * indicates a significant (*p* < 0.05/6) difference between the two conditions.

To investigate whether the BIC-evoked potential is related to listeners’ perception of the lead-lag fusion or not and which component of the potential is more closely related to the perception, we measured the reaction time for subjective detection of the BIC at different delays. This measurement was conducted in another group of subjects. As shown in Fig 8, the reaction time for subjectively detecting the BIC increased significantly with the delay [grey dots; F(3,30) = 88.604, *p* < 0.0001, one-way repeated ANOVA]. The increase of the reaction time already became significant when the delay was increased from 0 ms to 2 ms (*p* < 0.05/6 for all the six paired-sample t tests). In contrast, the reaction time for detecting the break in energy (squares) or the onset of the lead-lag pair (black dots) was not affected by the delay. Therefore, the reaction time for subjective detection of the BIC (Fig 8) and the peak latency of the P2 evoked by the BIC (Fig 6B) were similarly modulated by the delay. These results suggest that the BIC-evoked potential is related to listeners’ perception of the lead-lag fusion and that the P2 is more closely related to the perception than the N1.

## Discussion

This study investigated cortical correlates of the lead-lag fusion by introducing a BIC in the temporal middle of the lead-lag pair. We demonstrated that the BIC could evoke a cortical potential consisting of an N1 and a P2 component. Both components were modulated by the time delay between the lead and lag. The delay similarly modulated the peak amplitude of the N1 and the P2, an increase of the delay resulting in a decrease of the amplitude. In contrast, the delay differently modulated the peak latency of the two components, an increase of the delay resulting in an increase of the P2 latency but not an increase of the N1 latency. Similar to the P2 latency, the reaction time for subjective detection of the BIC increased with the delay. These findings suggest that both the N1 and the P2 evoked by the BIC are neural correlates of the lead-lag fusion and that, relative to the N1, the P2 may be more closely related to listeners’ perception of the fusion.

Introducing a BIC in the temporal middle of a lead-lag pair does not change the energy or the spectrum of the noises but changes the diffuseness, loudness, and pitch of the perceived noise image [9, 22–25]. Because of these perceptual changes, an auditory event separate from the ongoing noise can be perceived at the time when the BIC occurs. Perception of the BIC is therefore due to a change in the perceptual fusion between the lead and lag. It can be measured subjectively by asking listeners to report their perception [7, 8, 11, 13–16] or objectively by recording the cortical potential evoked by the BIC [18]. Both the subjective perception of the BIC and the objective cortical potential evoked by the BIC are modulated by the delay and by the center frequency of the noises [7, 11, 18]. Therefore, the objective cortical potential evoked by the BIC, especially the P2 component, can be utilized to estimate the subjective perception of the lead-lag fusion. Our study thus provides a potential approach to measure the strength of the perceptual lead-lag fusion on the populations having difficulty in giving subjective reports.

When the delay is 0 ms, a 200-ms BIC, as utilized in this study, is well above listeners’ detection threshold [11] and can evoked a cortical potential. This BIC-evoked potential is due to a stimulus-driven process rather than due to subjects’ temporal expectation (Fig 7). Nevertheless, the BIC-evoked potential was sluggish in time relative to the onset-evoked potential (Figs 3 and 4). This observation is consistent with the idea that the perception of the BIC requires additional processes such as neural computation of the correlation between the lead and lag across frequency channels and integration of the instantaneous computation results over time across these channels [12]. The neural computation of the correlation may start in brainstem structures like inferior colliculus and auditory thalamus [26–31]. The integration of the computation results has been suggested to occur in non-primary auditory cortex like the lateral part of Heschl’s gyrus and planum temporale [24, 26] and can result in perceptual features of the sound images such as diffuseness, loudness and pitch [12].

Relative to the N1, the P2 evoked by the BIC may be more closely related to the perceptual features of the sound images resulted from the lead-lag fusion. When the delay is > 0 ms, the fine structure information of the lead needs to be stored in “primitive” auditory memory for the neural computation of the correlation between the lead and lag as well as for the integration of the computation results and the stored information dissipates fast in time [4, 6–8, 11, 13, 30]. The dissipation leads to a de-correlation of the lead and lag at the neural level and thus a decrease of the perceptual contrast between the BIC and the ongoing noise marker. Therefore, in our study, when the delay was increased from 0 ms to 4 ms, the perceptual contrast between the BIC and the ongoing noise marker became smaller, which was reflected in the decrease of the amplitude of both the N1 and the P2 evoked by the BIC. Nevertheless, only the latency of the P2 increased with the delay while the latency of the N1 did not change. Because the delay modulated two features of the BIC-evoked P2 (i.e., both the amplitude and the latency) but only one feature of the N1 (i.e., only the amplitude), the P2 was more sensitive to the delay than the N1. We therefore speculate that, relative to the N1, the BIC-evoked P2 is more closely related to the late stages of the lead-lag fusion, i.e., more closely related to listeners’ perception of sound images. If this were the case, we would expect a close relation between the neural activity related to the late stages of the lead-lag fusion and listeners’ subjective perception of the fusion. Indeed, such a relation was observed in the way that the latency of the BIC-evoked P2 and listeners’ reaction time for subjective detection of the BIC increased similarly with the delay.

The cortical potential evoked by the BIC was clearly visible when the delay was 0 ms or 2 ms and became small when it was 4 ms. This longest delay of 4 ms for objective detection of the BIC in the EEG experiments is shorter than the longest delay of ~10 ms for subjective detection of the BIC in the psychoacoustic experiments using identical stimuli [7]. One reason for this difference could be the experimental paradigm. In the psychoacoustic experiments, we used a two-alternative-forced choice paradigm in which the BIC was the target for detection and subjects were required to make a choice in each trial. In the EEG experiments, we used a paradigm in which the target for detection was not the BIC itself, but instead was another auditory event (i.e., the 200-ms break in energy), and subjects were required to respond only in some of the trials. These different paradigms may result in different levels of attention on the stimuli and thus different longest delays for detecting the BIC in the two types of experiments.

## Conclusions

In this study, we measured human cortical potentials evoked by a BIC in the temporal middle of a lead-lag pair at different delays as well as the reaction time for subjective detection of the BIC. We observed effects of the delay on both the amplitude and the latency of the BIC-evoked potential. The delay affected the amplitude of both the N1 and the P2, suggesting that both components are neural correlates of the lead-lag fusion. The delay affected the latency of the P2 but not the latency of the N1. The effect of the delay on the P2 latency was similar to the effect of the delay on the reaction time for subjective detection of the BIC. Our findings therefore suggest that, relative to the N1, the P2 evoked by the BIC may be more closely related to listeners’ perception of the fusion.
